# Biological Activity and Stability of Aeruginosamides from Cyanobacteria

**DOI:** 10.3390/md20020093

**Published:** 2022-01-21

**Authors:** Marta Cegłowska, Patrycja Kwiecień, Karolina Szubert, Paweł Brzuzan, Maciej Florczyk, Christine Edwards, Alicja Kosakowska, Hanna Mazur-Marzec

**Affiliations:** 1 Institute of Oceanology, Polish Academy of Sciences, Powstańców Warszawy 55, PL-81712 Sopot, Poland; powczarczyk@iopan.gda.pl (P.K.); akosak@iopan.pl (A.K.); 2 Division of Marine Biotechnology, Institute of Oceanography, University of Gdańsk, M. J. Piłsudskiego 46, PL-81378 Gdynia, Poland; karolina.szubert@phdstud.ug.edu.pl; 3 Department of Environmental Biotechnology, Faculty of Geoengineering, University of Warmia and Mazury in Olsztyn, Słoneczna 45G, PL-10709 Olsztyn, Poland; brzuzan@uwm.edu.pl (P.B.); maciej.florczyk@uwm.edu.pl (M.F.); 4 School of Pharmacy and Life Sciences, Robert Gordon University, Aberdeen AB10 7GJ, UK; c.edwards@rgu.ac.uk

**Keywords:** cyanopeptides, aeruginosamides, cytotoxicity, miRNA, cytochrome P450 enzymes, metabolic stability

## Abstract

Aeruginosamides (AEGs) are classified as cyanobactins, ribosomally synthesized peptides with post-translational modifications. They have been identified in cyanobacteria of genera *Microcystis*, *Oscillatoria*, and *Limnoraphis*. In this work, the new data on the in vitro activities of three AEG variants, AEG A, AEG625 and AEG657, and their interactions with metabolic enzymes are reported. Two aeruginosamides, AEG625 and AEG657, decreased the viability of human breast cancer cell line T47D, but neither of the peptides was active against human liver cancer cell line Huh7. AEGs also did not change the expression of MIR92b-3p, but for AEG625, the induction of oxidative stress was observed. In the presence of a liver S9 fraction containing microsomal and cytosolic enzymes, AEG625 and AEG657 showed high stability. In the same assays, quick removal of AEG A was recorded. The peptides had mild activity against three cytochrome P450 enzymes, CYP2C9, CYP2D6 and CYP3A4, but only at the highest concentration used in the study (60 µM). The properties of AEGs, i.e., cytotoxic activity and in vitro interactions with important metabolic enzymes, form a good basis for further studies on their pharmacological potential.

## 1. Introduction

Cyanobacterial metabolites are frequently used as a starting material for the development of new drugs [1,2,3,4]. A derivative of dolastatin 10, a potent microtubule inhibitor produced by marine cyanobacterium *Symploca* (originally isolated from sea hare *Dolabella auricularia*), was approved for the treatment of Hodgkin’s lymphoma [5,6]. Largazole, another bioactive compound produced by *Symploca*, is explored as an anticancer drug that acts by inhibiting class I histone deacetylases (HDACs) [7]. Largazole crosses the blood-brain barrier and shows in vitro activity against glioblastoma multiforme cells [8]. Cyanovirin-N, a lectin produced by *Nostoc ellipsosporum*, acts as an antiviral agent. It prevents infection by blocking the interaction between human immunodeficiency virus (HIV) gp120 and the cell-associated CD4 receptor [9]. Another lectin, microvirin, shows similar activity, but has a simpler structure and better safety profile [10]. Unfortunately, not many of the cyanobacterial metabolites with promising bioactivity are leading molecules in drug development. One of the reasons is their poor pharmacokinetic profile. Therefore, in an early stage of hit-to-lead studies, the behaviour of the biomolecules under physiological conditions should be explored [11]. The determination of adsorption, distribution, metabolism, and elimination (ADME) is important in the case of all bioactive compounds to which people can be exposed through ingestion, injection, inhalation, or dermal contact.

Aeruginosamides (AEGs) are a group of linear cyanobactins, ribosomally synthesized and post-translationally modified peptides (RiPPs) produced by various cyanobacterial taxa [12]. The diversity of cyanobactins biosynthetic pathways generates a vast array of structures [13]. The most characteristic post-translational modification of the peptides includes the heterocyclisation of serine, cysteine, and threonine. The process is catalysed by heterocyclases and leads to the formation of thiazole, thiazoline, oxazole, or oxazoline [14,15]. The other common post-translational modification is the prenylation of Tyr, Ser, Thr, Trp, His, and the guanidine group in Arg, which is accomplished by different prenyltransferases [12,16,17,18,19]. Some cyanobactins, including patellamides and ulithiacyclamide, exhibit in vitro antitumor activity [20].

**Figure 1:**
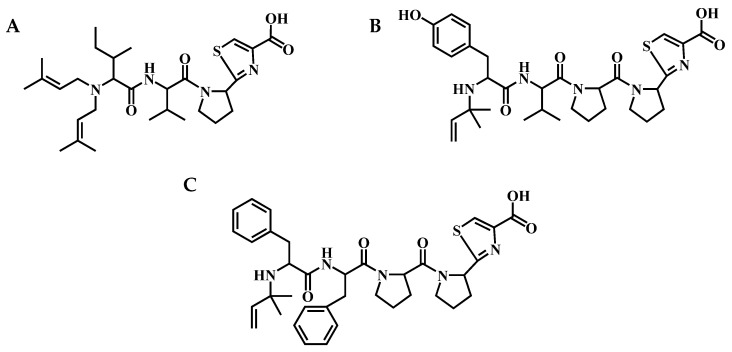
Structures of aeruginosamides: (**A**) AEG A, (**B**) AEG625, and (**C**) AEG657.

Existing knowledge about AEGs and their producers is limited. The first aeruginosamide, AEG A (Figure 1A), was discovered in *Microcystis aeruginosa*-dominated bloom sample from Rutland Water in England [21]. More recently, AEG B and AEG C were identified in *M. aeruginosa* PCC 9432 isolated from Little Rideau Lake in Ontario, Canada [12]. The aeruginosamide-like compound, called viridisamide A was identified in *Oscillatoria nigro-viridis* PCC 7112 from greenhouse soil in San Francisco, CA, USA [12]. The highest number of AEG variants (18) was detected in Baltic cyanobacterium *Limnoraphis* CCNP1324 [22]. AEG A was also obtained through chemical synthesis [23]. Aeruginosamides are composed of two to five amino acid residues, mainly Phe, Tyr, Ile, Val, and Pro [12,22]. They usually contain one or two prenyl groups at the *N*-termini and methyl ester of thiazolidine-4-carboxylic acid (TzlCOOMe) or thiazolidine-4-carboxylic acid (TzlCOOH) at the *C*-termini (Figure 1). In a previous study, AEG A showed cytotoxic activity against human ovarian tumour cells and human leukaemia cells [21], while AEG681a, isolated from Baltic *Limnoraphis* CCNP1324, reduced the viability of breast cancer cells [22].

In the current work, the pharmacological potential of aeruginosamides was explored. For this purpose, the in vitro activities of three isolated aeruginosamides, AEG A, AEG625 and AEG657 (Figure 1), against breast cancer cells (T47D) and liver cancer cells (Huh7) were tested. Luciferase reporter assays were used to examine whether AEGs act through the regulation of specific miRNA or through the induction of oxidative stress. In addition, to gain some knowledge on the possible behaviour of AEGs under physiological conditions, we studied the metabolic stability of peptides in the liver S9 fraction containing microsomal and cytosolic enzymes, and the effect of the AEGs on the activity of three cytochrome P450 enzymes CYP2C9, CYP2D6 and CYP3A4.

## 2. Results

### 2.1. Biological Activity

**Figure 2:**
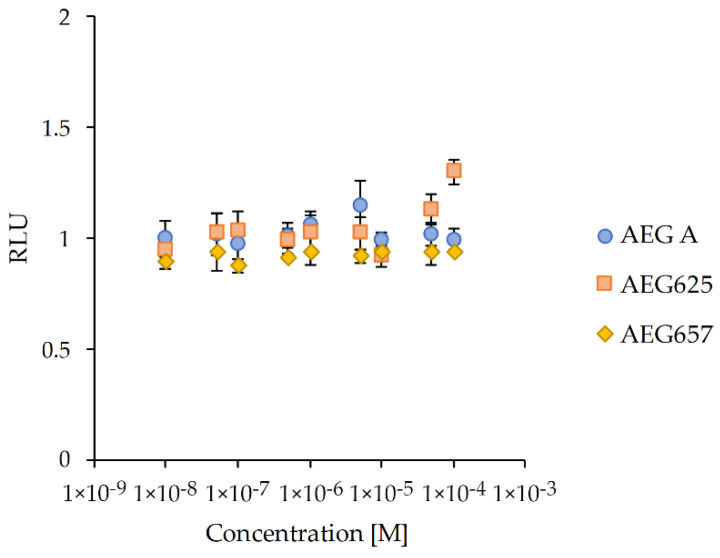
Luciferase assay response curves for Huh7 reporter cells exposed for 24 to increasing concentrations of the three AEGs. Relative luciferase activity was calculated using the ratio of firefly (F) and Renilla (R) luciferase activity (F/R) and was expressed in relative luminescence units (RLU). All assays were performed in triplicate in a 96-well format and were normalized to luciferase expression of cells treated only with the transfection reagent. Data are expressed as means with a standard deviation.

**Figure 3:**
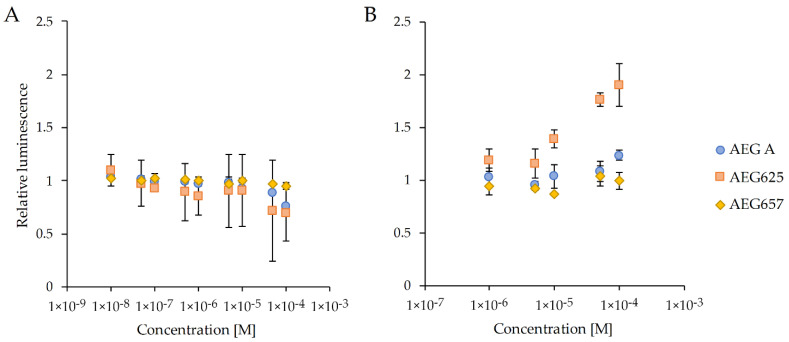
(**A**) Cell viability and (**B**) hydrogen peroxide levels in Huh7-donor cells exposed to increasing concentrations of AEGs. Luminescence values were normalized to untreated cells. All assays were conducted for 24 h in triplicate in a 96-well format. Data are expressed as means with a standard deviation.

AEG625 (Figure 1B) and AEG657 (Figure 1C) were isolated (4.582 and 1.15 mg, respectively) from Baltic cyanobacterium *Limnoraphis* strain CCNP1324, and AEG A (60 mg) was obtained from the *Microcystis* bloom sample. The cytotoxicity of the three peptides was tested using breast cancer cell line T47D (Figure S1). Two of the peptides, AEG625 and AEG657, at a concentration of 40 µM, inhibited T47D viability by 48% (SD = 6) and 63% (SD = 8), respectively. In the case of AEG A, no effects were observed. The three AEGs were also tested for activity towards a specific miRNA, MIR92b-3p, expressed in human hepatoma cell line Huh7. In the reporter assay, the cyanopeptides did not restore the luciferase signal in Huh7 cells stably transfected with a pmirGLO vector containing dual-luciferase reporters (Huh7-pmirGLO-92b cells). Only exposure to 100 µM of AEG625 caused a slight increase in signal intensity (Figure 2). AEG625 also had no apparent effect on Huh7 cell viability, analysed using CellTiter-Glo 2.0 Cell Proliferation Assay. However, at the highest concentrations (50 and 100 µM), a slight drop in mean luciferase expression was observed (0.73 (SD = 0.47) and 0.70 (SD = 0.27) of relative luminescence, respectively; Figure 3A), which suggested lowered cell survival. In the third reporter assay, using a ROS-Glo™H_2_O_2_ Assay Kit, only AEG625 altered ROS (reactive oxygen species) levels (twofold) in Huh7 cells in a concentration-dependent manner (Figure 3B).

### 2.2. Metabolic Stability in S9 Fraction

**Figure 4:**
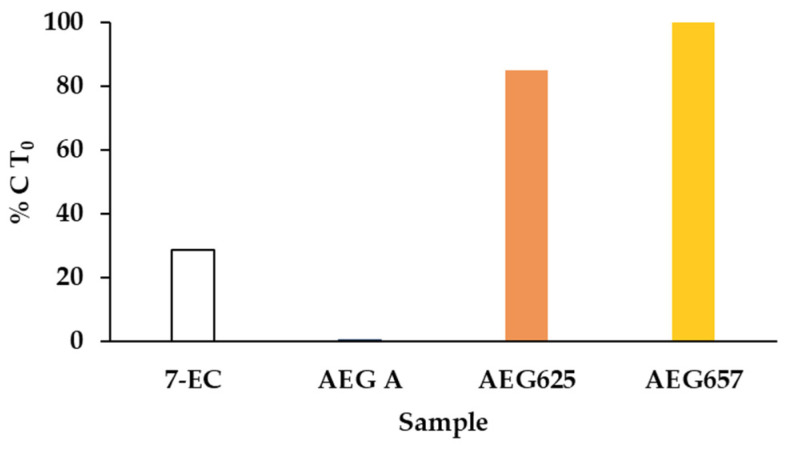
Concentrations of 7-EC, AEG A, AEG625, and AEG657 after 60 min exposure to S9 fraction containing microsomal and cytosolic enzymes. Results are expressed in relation to initial concentration of the compounds (%C T_0_).

The metabolic stability of the three AEGs in liver S9 fraction containing microsomal and cytosolic enzymes was determined by LC-MS/MS. To monitor the catalytic activity of the enzymes, 7-etoxycoumarin (7-EC) was used as a positive control, while the sample with heat-denatured S9 fraction served as a negative control. During the assay, a systematic decrease in the concentration of 7-EC exposed to the S9 fraction was recorded (Figure 4). After 60 min, the concentration dropped to 28.6% (SD = 6.8) of the initial value (elimination half-life t_1/2_ = 38.7 min). At the same time, no changes were observed in the heat-denatured S9 fraction. For aeruginosamides AEG657 and AEG625, neither changes in the active nor in the deactivated fraction were detected. In contrast, the mixture of enzymes contained in the S9 fraction caused a rapid decrease in AEG A concentration (t_1/2_ = 2.48 min). After 15 and 60 min, only 2.9% (SD = 2.05) and 0.4% (SD = 0.4) of the initial AEG A were detected (Figure 4 and Figure S2A). A rapid decrease in AEG A concentration was also observed in the heat-denatured S9 fraction. After 15 and 60 min, 56.0% (SD = 0.94) and 46.0% (SD = 9.76) of the initial AEG A concentrations were measured (Figure S2B).

### 2.3. Inhibition of Cytochrome P450 Enzymes

**Figure 5:**
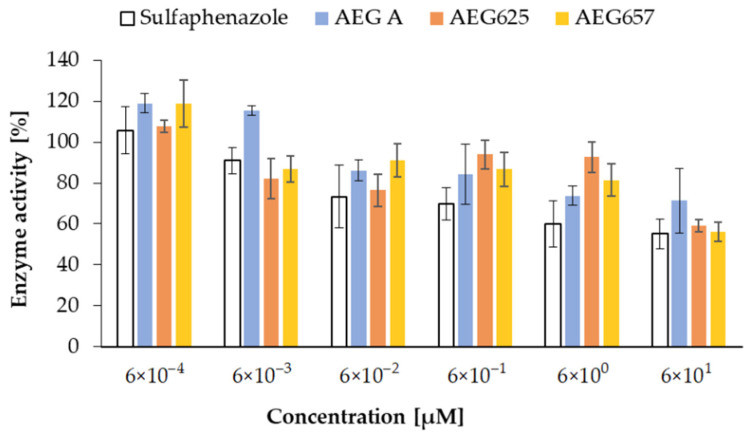
Effects of AEG A, AEG625, and AEG657, applied at different concentrations, on activity of CYP2C9 human P450 enzyme (after 60 min incubation). Data are expressed as means with a standard deviation.

The effect of the three aeruginosamides on cytochrome P450 was studied with the application of Vivid^®^ CYP450 Screening Kits with baculovirus-expressed human P450 enzymes CYP2C9, CYP2D6, and CYP3A4. The activity of the tested compounds was evaluated on the basis of changes in fluorescent properties of the Vivid^®^ fluorogenic substrates. All tested AEGs inhibited CYP2C9 activity in a concentration-dependent manner (Figure 5). The effects were moderate and comparable to sulfaphenazole (IC_50_ = 6.1 µM), the standard CYP2C9 inhibitor. At the highest concentration (60 µM), sulfaphenazole reduced CYP2C9 activity by 44.9% (SD = 7.2), while enzyme activity in the presence of AEG A, AEG657 and AEG626 was reduced by 28.7% (SD = 15.8), 40.7% (SD = 4.6), and 43.9% (SD = 2.9), respectively (Figure 5). Quinidine, the standard inhibitor of CYP2D6 (IC_50_ = 4.7 µM), when used at the two highest concentrations (6 and 60 µM), decreased the activity of the enzyme by 58.3% (SD = 2.5) and 96.2% (SD = 1.9). The three aeruginosamides revealed weaker activity against the enzyme than the standard (Figure S3A). Of the three peptides, the most pronounced effect was observed in the case of AEG A (IC_50_ = 8.6 µM), which at the two highest concentrations reduced the activity of CYP2D6 to 78.4% (SD = 1.6) and 60.4% (SD = 2.8) (Figure S3A). AEG657 was active only at a concentration of 60 µM (inhibition by 33.2%; SD = 0.0), while AEG625 did not induce any effects. Ketoconazole (standard) inhibited CYP3A4 in a concentration-dependent manner. When the concentration of the compound changed from 0.06 to 60 µM, activity decreased from 67.6% (SD = 6.0) to 4.7% (SD = 2.7) (IC_50_ = 3.3 µM) (Figure S3B). AEG A was active against CYP3A4, but only at the two highest concentrations used in the assay (IC_50_ = 5.7 µM) (Figure S3B). In the presence of AEG657, the activity of the enzyme remained stable and even at 60 µM, it was only by approx. 18% lower than that in the control. No significant loss of CYP3A4 activity was observed in the presence of AEG625 (Figure S3B).

## 3. Discussion

Cytotoxicity is the most commonly reported type of activity of cyanobacterial metabolites as recognized in approximately 34% of the tested compounds [2]. They were mainly isolated from the representatives of the *Lyngbya*, *Symploca*, *Anabaena*, *Tolypothrix*, *Nostoc* and *Phormidium* genera, and belong to depsipeptides, lipopeptides, macrolides, and nucleosides [2,24,25]. The cytotoxic activity of cyanobactins, including their linear variants classified to aeruginosamides, was also reported [20,21,22]. In our current study, the cytotoxic activity of three aeruginosamides, AEG A, AEG625, and AEG657, was explored. The structure of AEG A was elucidated by Lawton et al. [21] on the basis of nuclear magnetic resonance (NMR) and mass spectrometry (MS) analyses. The structures of AEG625 and AEG657, together with 16 other aeruginosamides, were characterized by Cegłowska et al. [22] on the basis of the product ion spectra of their pseudomolecular ions. For one of the peptides (AEG671, inactive against T47D), the correctness of structure elucidation was confirmed by NRM [22]. Cytotoxicity assays revealed in vitro activity of two aeruginosamides, AEG625 and AEG657, against breast cancer cell line T47D, and a lack of significant effects on liver cancer cell line Huh7. Only at the highest concentration of AEG625 (100 µM) a slight decrease in Huh7 viability was recorded. In contrast, AEG A, with the most different structure from the other two peptides, did not affect either of the two cancer cell lines. In a previous study, this aeruginosamide variant showed cytotoxic activity towards A2780 human ovarian tumour cells (IC_50_ = 2.9 µM) and K562 human leukaemia cells (IC_50_ = 5.2 µM) [21]. The three aeruginosamides used in our work contain one (AEG625 and AEG657) or two (AEG A) prenyl groups attached to the *N*-terminal amino acid [21,22]. Natural forward or revers prenylated compounds, or compounds with chemically substituted prenyl groups are more cytotoxic against cancer cells than their nonprenylated variants [26,27,28,29]. These effects are probably related to the fact that the prenyl group increases hydrophobic properties and enhances the permeability of the compounds through cellular membranes. The prenyl group also facilitates the affinity of compounds to proteins [27,30]. The activity of AEGs against cancer cells can depend on amino acid composition and their sequence. However, too few structural variants of the peptides were tested to draw conclusions on the significance of any specific residue and/or its position on the observed effects. The cell-line-dependent activity of different AEGs observed in our work also requires further studies. Cyanometabolites can control the proliferation and growth of cancer cells through different mechanisms [31,32]. At least some of the cytotoxic compounds could be RNA interference (RNAi) modulators, likely acting through the regulation of specific miRNAs. These small noncoding RNAs regulate the expression of various genes and play an important role in a wide range of cellular processes [33,34]. As the dysregulation of miRNA is linked to a number of diseases, including cancer, small molecules that interfere with miRNA are considered to be potential drug candidates [35,36]. In a previous study, a quantitative screening strategy to identify molecules that modulate miRNA 92b-3p expression in liver cells was developed [37]. The same screening strategy with Huh7 human hepatoma cell line stably transfected with a pmirGLO vector was used in current work to examine the effect of the three aeruginosamides on MIR92b-3p. Only for AEG625, which caused a slight but not significant decrease in Huh7 viability, an increase in reactive oxygen species level was recorded in the cells. Taken together, these results indicate that AEG625 had a weak, if any, effect on the expression of MIR92b-3p in the Huh7 cell line, but moderately affected the viability of Huh7 cells, most likely due to ROS generation.

This change in reactive oxygen species could probably be neutralized by the natural cell defence system against oxidative stress. The disruption of redox homeostasis can damage DNA, proteins, and lipids [38]. On the other hand, cancer cells have enhanced sensitivity to oxidative stress, and ROS-inducing agents are applied in anticancer therapies [39,40,41]. Therefore, the activity of AEG625 does not need to exclude the peptide from the list of lead candidates. AEG625 differs from AEG657 with respect to two residues: the *N*-terminal Tyr-Val in AEG625 is replaced by Phe-Phe in AEG657. Apparently, this peptide fragment determines the ROS-inducing activity of the compounds.

All natural products that show in vitro activity against drug targets should be evaluated in terms of their pharmacokinetic properties. The early detection of unfavourable properties of the bioactive compounds, preferably at the hit-to-lead stage, saves the efforts and costs related to drug development [42]. On the basis of the results of the high-throughput assays, the numerous natural products detected in biological samples can be prioritized for further studies. The tests can also supply valuable information that can be used to optimise the structure of bioactive agents. As all the tested AEGs showed cytotoxic effects against at least one cancer cell line, further in vitro experiments were performed to gain some insight into their pharmacological potential. The stability of AEGs in the liver S9 fraction containing both microsomal and cytosolic enzymes was analysed. The catalytic activity of the enzymes requires the presence of specific cofactors [43]. For this purpose, in our study, β-nicotinamide adenine dinucleotide phosphate (NADPH, Phase I metabolism), uridine 5′-diphospho-α-d-glucuronic acid (UDPGA, Phase II metabolism) and glutathione (GSH, Phase II metabolism) were used. AEG625 and AEG657 showed high metabolic resistance. The stability of the peptides can be attributed to the presence of proline residues, which allow for peptides and proteins to be less susceptible to proteolytic attack [44]. In the case of AEG A, within the 60 min of the experiment, significant time-dependent depletion was recorded. The faster degradation of the bis-prenylated AEG A than that of the two other AEGs may be attributed to the loss of one of the two prenyl groups located at the *N*-terminal residue of AEG A. Loss of the group was also determined by LC-MS/MS during longer storage of the peptide in aqueous methanol solution. The other possible reason for a rapid decrease in the concentration of hydrophobic AEG A (with two prenyl groups) is its ability to bind to proteins in the S9 fraction. The proteins were removed from the sample prior to the quantification of the peptide by LC-MS/MS. The reduction in AEG A in the presence of the heat-denatured S9 fraction indicates the contribution of the nonenzymatic pathway to the changes.

In the second step, the Vivid^®^ CYP450 Screening Assay Kit [45] was used to determine the effect of AEGs on the activity of the three individual, heterologously expressed cytochrome P450 enzymes CYP2C9, CYP2D6 and CYP3A4. Cytochrome P450, the superfamily of enzymes, is involved in the metabolism of endogenous and exogenous compounds, including drugs and xenobiotics [46,47]. The role of CYP450 and their substrate specificity is different. The highest number of drugs (15–58%) is metabolised by members of the CYP3A family, while CYP2C and CYP2D6 are involved in the metabolism of 8–35% and 0.1–6% of the compounds, respectively [39,40]. The activity of the enzymes is also modified by different inhibitors. For example, quinidine is the standard inhibitor of CYP2D6, and sulfaphenazole is selectively active against CYP2C9. Ketoconazole belongs to CYP3A4 inhibitors, but it also inhibits the activity of other cytochrome P450 enzymes [48,49]. In addition, natural products, such as coumarin, flavonoids, nicotine, aflatoxin, estradion, and ginger, can have an inhibitory effect on the enzymes [50,51]. The inhibitory activities of aeruginosamides used in our work were rather moderate and only observed at the highest concentrations. AEG A was most active towards CYP3A4, but its effects on CYP2D6 and CYP2C9 were weak. AEG625 slightly inhibited only CYP2C9, and did not affect the two other enzymes. AEG657 had no significant effect on CYP3A4, and showed only weak inhibition against CYP2C9 and CYP2D6. Neither of the peptides was a stronger inhibitor than the standard inhibitors used in the assay. P450 enzymes are the most important components of Phase I metabolism; their inhibitors increase the risk of drug–drug interaction and negatively impact the biotransformation of xenobiotics. Thus, the low activity of AEGs against the enzymes observed in our assays could indicate a lack of adverse effects of the peptides on these processes. If any of the peptides reveal pharmacologically important activity against some biological targets, this finding can increase the chances of the compound to be classified as a lead candidate in the drug development process.

Generally, there is considerable interest in peptide-based therapeutic agents, for example, as anticancer peptides (ACPs) [52,53]. These compounds have many beneficial properties, and their synthesis for commercial purposes is relatively easy and cost-effective. Moreover, modifications introduced to the structure can lead to improvement in their drug-like properties. Cyclic forms usually show greater potential than the linear ones [54]; however, linear pentapeptide dolastatin-10 from marine cyanobacterium *Symploca* was successfully developed into an anticancer agent [55,56]. In this work, we report new findings on the in vitro biological activity of the three aeruginosamides, AEG A, AEG625 and AEG657, and on their interactions with important metabolic enzymes. Results illustrate the impact of chemical structure on the biological activity of the compounds. Although the peptides belong to the same class and share common elements, significant differences in their activity and stability were recorded. With respect to these properties, AEG A with two prenyl groups and a shorter peptide chain was the most different than AEG625 and AEG657. It did not affect T47D, and its stability in the S9 assay was significantly lower. The results of the in vitro assay may have low correlation with the in vivo behaviour of aeruginosamides, as revealed in the case of other compounds [57]. Nonetheless, in conjunction with findings from other analyses, they can provide an important indication about the peptides as possible lead molecules in drug development. In the current study, three AEGs were tested only against selected cell lines. For more complete recognition of their possible therapeutic application, activity against a wider array of cell targets should be determined.

## 4. Materials and Methods

### 4.1. Extraction and Isolation of Aeruginosamides

Cyanobacterium *Limnoraphis* sp. CCNP1324 was grown for biomass in F/2 medium (7 PSU) [22]. The freeze-dried material (20 g) was homogenised and extracted twice with 75% methanol (MeOH) in water (*v*/*v*) by vortexing. The combined extracts (1 L) were centrifuged (10,000× *g*; 15 min; 4 °C) and diluted with Milli-Q water, so that the final concentration of methanol in the samples was <10%. Components of the extract were separated using a Shimadzu HPLC system (Shimadzu Corporation, Kyoto, Japan) with a photodiode array detector. Absorbance was measured at 210 and 280 nm. The sample was loaded onto a preconditioned 120 g SNAP KP-C_18_-HS column (Biotage, Uppsala, Sweden); then, compounds were eluted with a mixture of water and methanol, where the content of methanol changed from 10% to 100% over 120 min. Flow rate was 15 mL min^−1^, and 45 mL fractions were collected.

Fractions containing the selected AEG as a dominant component were combined; then, the organic solvent was removed in a centrifugal vacuum concentrator (MiVac, SP Scientific, Ipswich, UK) at 30 °C. Each fraction was first loaded onto a SecurityGuard^TM^ PREP Cartridge (C_12_; 15 × 30 mm) (Phenomenex, Torrence, CA, USA). Then, chromatographic separation was performed in a Jupiter Proteo C_12_ column (250 × 21.2 mm; 4 µm; 90 Å) (Phenomenex, Torrence, CA, USA) with a mobile phase composed of 5% acetonitrile in water (A) and acetonitrile (B), both with the addition of 0.1% formic acid. Flow rate was 15 mL min^−1^, and 4 mL fractions were collected. The gradient elution program started at 15% and went to 50% B in 50 min, then to 100% B in the next 20 min.

AEG A was isolated and purified as described by Lawton et al. [21]. In brief, frozen bloom material (4 L of concentrated scum; approximately 128 g wet weight), dominated by *Microcystis aeruginosa*, was extracted 3 times with MeOH following thawing. Pooled extracts were diluted to 20% MeOH, filtered and loaded onto a flash chromatography column (C_18_ column, 40 mm × 15 cm; Biotage, Cardiff, UK). The cartridge was eluted by a step gradient of 0–100% MeOH in 10% increments. AEG A was eluted in the 90% MeOH fraction and required decolourisation by a second flash chromatography separation.

### 4.2. Luciferase Reporter Assays

In this study, the reporter assays for small-molecule inhibitors of the MIR92b-3p function, previously designed to screen *Nostoc* peptides, were used [37]. The screen employs a Huh7 human hepatoma cell line (Cell Lines Service GmbH, CLS; Eppelheim, Germany) stably transfected with a pmirGLO vector containing dual-luciferase reporters. Briefly, for the chemical screening of AEGs, Huh7-pmirGLO-MIR92b-3p cells were seeded at 10,000 cells per well in white, 96-well plates (Thermo Fisher Scientific, Waltham, MA, USA). After overnight incubation, the medium was removed and replaced with 100 μL of RPMI1640 (Merck, KGaA, Darmstadt, Germany), supplemented with increasing concentrations of AEGs, ranging from 10 nM to 100 μM, with three replicates at each concentration. Cells were incubated for 24 h and then analysed with a dual-luciferase Reporter Assay Kit (Promega, Madison, WI, USA). Relative luciferase activity was calculated using the ratio of firefly (F) and Renilla (R) luciferase activity (F/R).

To determine cell viability and ROS level, Huh7 cells were seeded at 10,000 cells per well in white 96-well plates. After overnight incubation, the medium was removed and replaced with 100 μL of RPMI1640 supplemented with increasing concentrations of AEGs ranging from 10 nM to 100 μM (cell viability) or ranging from 1 µM to 100 μM (ROS), with three replicates at each concentration. After incubation for 24 h, cells viability was examined using a Cell Titer-Glo Luminescent Cell Viability Assay (Promega, Madison, WI, USA) and ROS was measured using a ROS-Glo H_2_O_2_ Assay (Promega, Madison, WI, USA).

### 4.3. MTT Assay

MTT (3-(4,5-dimethylthiazol-2-yl)-2,5-diphenyltetrazolium-bromide) assay was performed according to Felczykowska et al. [58]. Briefly, cytotoxicity assessment was conducted against human breast adenocarcinoma cell line T47D obtained from the European Collection of Authenticated Cell Cultures (ECACC) through Merck KGaA (Darmstadt, Germany). Cells were cultured in 96-well plates using RPMI1640 medium (Carl Roth GmbH, Karlsruhe, Germany) supplemented with a fetal bovine serum (10% *v*/*v*; Sigma-Aldrich by Merck KGaA, Darmstadt, Germany) and penicillin-streptomycin solution (1% *v*/*v*, stock solution 50 u and 0.05 mg mL^−1^, respectively; Merck KGaA, Darmstadt, Germany) at 1 × 10^4^ cells per well and incubated for 24 h. After the addition of the tested compounds (40, 80, 160 and 320 µM) dissolved in 1% DMSO, samples were incubated for another 24 h. Then, MTT reagent was added (25 µL at 4 mg mL^−1^ in PBS; Merck KGaA; Darmstadt, Germany) and the samples were incubated for 4 h. Lastly, formazan crystals were dissolved in 100% DMSO and absorbance was measured using a microplate reader (Spectramax i3, Molecular Devices, LLC San Jose, CA, USA). Cell viability was calculated as the ratio of the mean absorbance value measured in two independent assays, for the three replicates containing the samples, to the mean absorbance of the three replicates of the corresponding solvent control and expressed as a percentage.

### 4.4. In Vitro S9 Stability Assay

Human S9 liver fraction (catalogue no. HMS9PL, lot no. PL045-A) (protein content 20 mg mL^−1^), containing a mixture of microsomal and cytosol enzymes, was used (Thermo Fisher Scientific, Waltham, MA USA) together with the following activating cofactors: NADPH (Alfa Aesar, Wart Hill, MA, USA), UDPGA (Merck KGaA; Darmstadt, Germany) and GSH (Merck, Darmstadt Germany). All cofactors (20 mM) and the analysed AEGs (3 µM) were prepared in 1% DMSO, the reaction was performed in a buffer (200 mM Tris (Merck KGaA, Darmstadt, Germany), 2 mM MgCl_2_ and 1M NaOH (Avantor Performance Materials Poland S.A., Gliwice, Poland); pH 7.4) 7-etoxycoumarine (7-EC; 3 µM in 20% DMSO) (Merck KGaA, Darmstadt, Germany) was used as a positive control. Tubes with 236 µL of buffer and 24 µL of S9 fraction were prepared in duplicates. Half of the samples was kept on ice, while the other half was deactivated by heating at 80 °C for 10 min and then cooled to room temperature. Cofactors were mixed (1:1:1) and 120 µL of the mixture, together with 20 µL of 7-EC were added to all samples. The aliquots (25 µL) of the samples were withdrawn at 0, 15, 30, 45, and 60 min, and 50 µL of an ice-cold ACN:MeOH (50:50) was added to stop the reaction. The samples were centrifuged (10,000× *g*, 4 °C, 10 min) and the supernatants were analysed by LC-MS/MS. The test was repeated twice. The elimination half-life was calculated as in Wen and Walle [59].

### 4.5. Cytochrome P450 Enzyme Inhibition Assays

The following commercially available Vivid^®^ CYP450 Screening Kits were performed according to the instruction supplied by the manufacturer (Thermo Fisher Scientific, Waltham, MA, USA): Vivid^®^ CYP2C9 Blue (cat. no. P2861), Vivid^®^ CYP2D6 Blue (cat. no. P2972) and Vivid^®^ CYP3A4 Blue (cat. no. P2857). The CYP450 BACULOSOMES Plus Reagent (with the recombinant human cytochrome P450 and human cytochrome 450 reductases), Vivid Regeneration System (333 mM glucose-6-phosphate and 30 U mL^−1^ glucose-6-phosphate dehydrogenase in 100 mM K_3_PO_4_, pH 8.0), and Vivid NADP^+^ (10 mM NADP^+^ in 100 mM K_3_PO_4_, pH 8.0) were thawed at room temperature and kept on ice until use. After adjustment to room temperature, the Vivid substrate was dissolved in anhydrous acetonitrile (10 µM), and the fluorescence standard was dissolved in DMSO (100 µM). The test compounds and inhibitors were diluted with the Vivid^®^ CYP450 reaction buffer. As inhibitors, sulfaphenazole (for CYP2C9; at 30 µM), quinidine (for CYP2D6; at 10 µM) and ketoconazole (for CYP3A4; at 10 µM) all from Merck (KGaA, Darmstadt, Germany) were used. In the subsequent steps, 5 µL solutions of test compounds, inhibitors and solvent were added to the wells. Then, 5 µL of Master Pre-Mix (mixture of Reagent, Regeneration System and the Reaction Buffer) was dispensed to each well. After 10 min incubation at room temperature, the reaction was started by the addition of 5 µL of a mixture composed of Vivid^®^ Substrate and NADP^+^. Tests were performed in endpoint mode using 384-well plates. Excitation wavelength was 415 nm and emission was measured at 460 nm, at the start of the test and after 60 min. The test was performed with three replicates at each concentration. The percentage inhibition by the AEGs was calculated as suggested in the protocol provided by the kit supplier.

### 4.6. LC-MS/MS Analysis

LC-MS/MS analyses were performed using an Agilent 1200 (Agilent Technologies, Waldboronn, Germany) chromatographic system coupled with a hybrid triple quadrupole/linear ion trap mass spectrometer (QTRAP5500, Applied Biosystems, Sciex, Concorde, ON, Canada) as a detector. AEGs were separated in a Zorbax Eclipse XDB-C_18_ column (4.61 mm × 150 mm; 5 µm) (Agilent Technologies, Santa Clara, CA, USA). The composition of the mobile phase was the same as in preparative chromatography. For analysis of the collected chromatographic fractions, the gradient changed from 15% B to 99% B over 20 min (flow rate 0.6 mL min^−1^). The Turbo IonSource operated in positive ionisation mode at capillary voltage of 5.5 kV and source temperature of 550 °C. Cone gas pressure and desolvation gas pressure were 60 psi, declustering potential was 80 V, and collision energy was 60 ± 20 eV. Q3-information-dependent analysis (IDA) and enhanced product ion (EPI) modes were used. The IDA threshold was set at 500,000 cps, and the *m/z* range was 400–1000. The scan rate was 2000 amu s^−1^, and the linear ion trap fill time 50 ms. For analysis of AEGs and 7-EC, the chromatographic gradient changed from 15% B to 25% B in 5 min, then to 99% B in the next 5 min (0.6 mL min^−1^). MS operated in multiple reaction monitoring mode at collision energy CE 50 V and with the following transitions: 191 → 163, 119, 107 (q), 91 (for 7-EC); 561 → 222, 186, 86 (q) (for AEG A); 626 → 296, 136 (q), 70 (for AEG 625) and 658 → 296, 199 (q), 120 (for AEG 657). To determine the changes in AEGs concentrations, the peak areas of the most intensive ions (q) were measured.

## Data Availability

Not applicable.

## Supplementary Materials

The following supporting information can be downloaded at https://www.mdpi.com/article/10.3390/md20020093/s1. Figure S1: The effects of aeruginosamides AEG A (from *Microcystis* bloom sample), AEG625 and 657 (from *Limnoraphis* sp. CCNP1324), used at a concentration of 40 µM, on the viability of T47D human breast cancer cells assayed by MTT method. Data are expressed as means with a standard deviation. Figure S2: Reduction in 7-EC and AEG A concentrations after 60-min exposure to (A) active and (B) inactive S9 fraction containing microsomal and cytosolic enzymes. The results are expressed in relation to the initial concentration of the compounds (%C T_0_). Figure S3: Effects of AEG A, AEG625 and AEG657, applied at different concentrations, on the activity of (A) CYP2D6 and (B) CYP3A4 human P450 enzymes (after 60-min incubation).
