# Supplementary material for: Biological Activity and Stability of Aeruginosamides from Cyanobacteria
Source: Mar Drugs. 2022 Jan 21;20(2):93. doi: 10.3390/md20020093 (PMC8878463; doi:10.3390/md20020093)
Supplement: Supplementary file 1 [file marinedrugs-20-00093-s001.zip › supplementary.pdf]

marine drugs

MDPI

Article

Biological activity and stability of aeruginosamides from cyanobacteria

Marta Cegłowska, Patrycja Kwiecień, Karolina Szubert, Paweł Brzuzan, Maciej Florczyk, Christine Edwards, Alicja Kosakowska, Hanna Mazur-Marzec

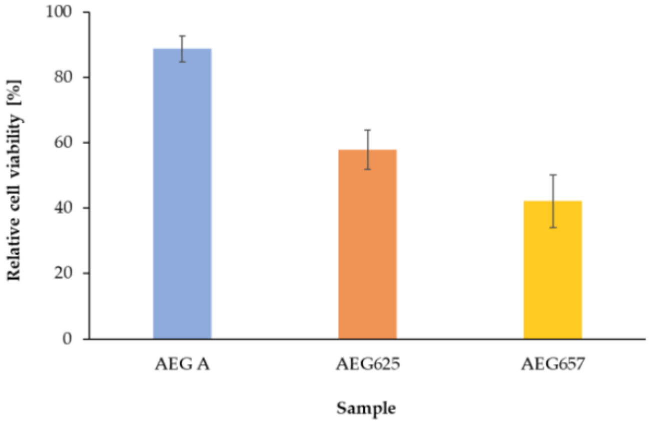

Figure 1. The effects of aeruginosamides AEG A (from Microcystis bloom sample), AEG625 and AEG657 (from Limnoraphis sp. CCNP1324), used at a concentration of 40 μM, on the viability of T47D human breast cancer cells assayed by MTT method. Data are expressed as means with a standard deviation.

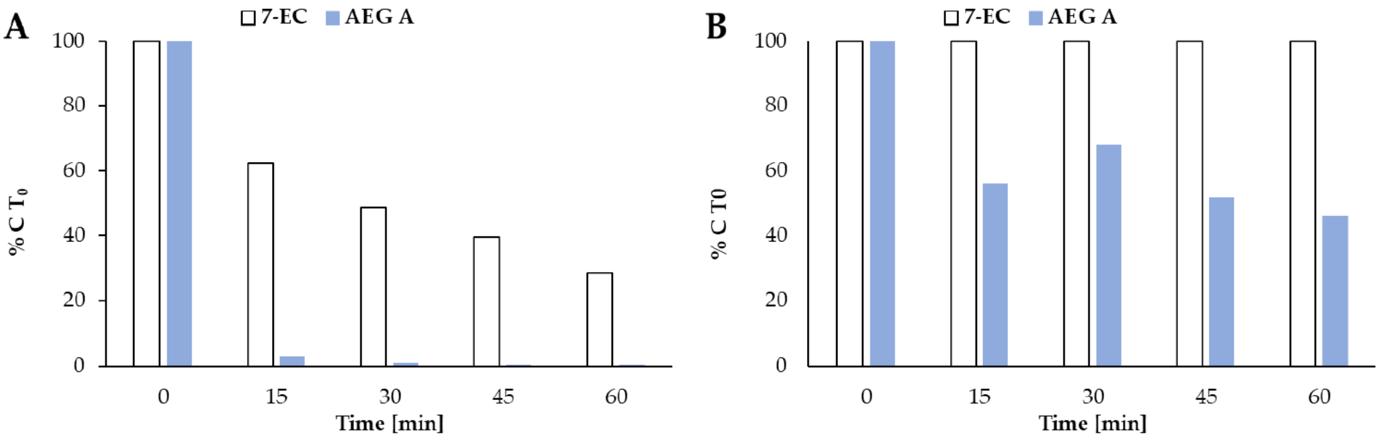

Figure 2. Reduction in the 7-EC and AEG A concentrations after 60-min exposure to (A) active and (B) inactive S9 fraction containing microsomal and cytosolic enzymes. The results are expressed in relation to the initial concentration of the compounds (%C T_0).

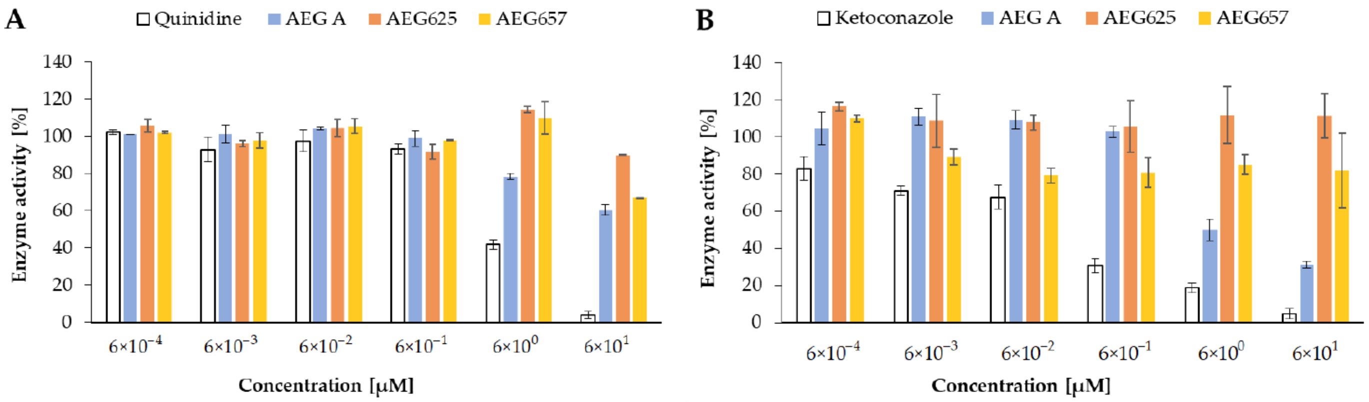

Figure 3. Effects of AEG A, AEG625 and AEG657, applied at different concentrations, on the activity of (A) CYP2D6 and (B) CYP3A4 human P450 enzymes (after 60-min incubation). Data are expressed as means with a standard deviation.
